# Very low prevalence of germline *MSH6* mutations in hereditary non-polyposis colorectal cancer suspected patients with colorectal cancer without microsatellite instability

**DOI:** 10.1038/sj.bjc.6603478

**Published:** 2006-11-21

**Authors:** C M Kets, J H J M van Krieken, K M Hebeda, S J Wezenberg, M Goossens, H G Brunner, M J L Ligtenberg, N Hoogerbrugge

**Affiliations:** 1 Department of Human Genetics, Radboud University Nijmegen Medical Centre, 849 Human Genetics, PO Box 9101, 6500 HB Nijmegen, The Netherlands; 2 Department of Pathology, Radboud University Nijmegen Medical Centre, The Netherlands

**Keywords:** MSI, HNPCC, hereditary cancer, *MSH6*

## Abstract

Hereditary non-polyposis colorectal cancer (HNPCC) is caused by mutations in one of the mismatch repair genes *MLH1*, *MSH2*, *MSH6*, or *PMS2* and results in high-level microsatellite instability (MSI-high) in tumours of HNPCC patients. The MSI test is considered reliable for indicating mutations in *MLH1* and *MSH2*, but is questioned for *MSH6*. Germline mutation analysis was performed in 19 patients with an MSI-high tumour and absence of MSH2 and/or MSH6 protein as determined by immunohistochemistry (IHC), without an *MLH1* or *MSH2* mutation, and in 76 out of 295 patients suspected of HNPCC, with a non-MSI-high colorectal cancer (CRC). All 295 non-MSI-high CRCs were analysed for presence of MSH6 protein by IHC. In 10 patients with an MSI-high tumour without MSH2 and/or MSH6 expression, a pathogenic *MSH6* mutation was detected, whereas no pathogenic *MSH6* mutation was detected in 76 patients with a non-MSI-high CRC and normal MSH6 protein expression. In none of the 295 CRCs loss of MSH6 protein expression was detected. The prevalence of a germline *MSH6* mutation is very low in HNPCC suspected patients with non-MSI-high CRC. Microsatellite instability analysis in CRCs is highly sensitive to select patients for *MSH6* germline mutation analysis.

Hereditary non-polyposis colorectal cancer syndrome (HNPCC) is an autosomal-dominant inherited disorder predisposing to colorectal cancer (CRC) and several other cancers at an early age, including endometrial carcinoma. It is clinically suspected by Amsterdam criteria and Bethesda guidelines (Rodriguez-Bigas *et al*, 1997; Umar *et al*, 2004). Hereditary non-polyposis colorectal cancer is caused by mutations in one of the mismatch repair (MMR) genes (*MLH1*, *MSH2*, *MSH6*, and *PMS2*) and is characterised by tumours that show microsatellite instability (MSI). Failure of MMR results in MSI especially in short repetitive sequences. Molecular testing for HNPCC can be performed by testing tumours for MSI and absence of MLH1, PMS2, MSH2 and/or MSH6 as determined by immunohistochemistry (IHC), and germline mutation analysis of MMR genes.

In clinical practice MSI analysis is used as a prescreening tool to select families for further analysis of MMR gene defects. Germline mutations in the *MSH6* MMR gene account for approximately 15–30% of cases of HNPCC (Hampel *et al*, 2005; Barnetson *et al*, 2006; Niessen *et al*, 2006). However *MSH6* mutation carriers were reported to have tumours without an MSI-high pattern (Berends *et al*, 2002; Hendriks *et al*, 2004; Plaschke *et al*, 2004), whereas in *MLH1* and *MSH2* mutation carriers almost all HNPCC-associated tumours show MSI (Lynch and Lynch, 2005). The reliability of MSI analysis to select patients at risk for *MSH6* mutations is therefore questioned. As germline mutation analysis and IHC of MMR proteins is almost exclusively initiated when MSI analysis shows MSI, we might miss *MSH6* germline mutations.

The aim of this study was to establish the prevalence of *MSH6* mutations in HNPCC suspected patients without MSI in their tumours to investigate the value of MSI analysis to detect *MSH6* mutations.

## MATERIALS AND METHODS

**Figure 1:**
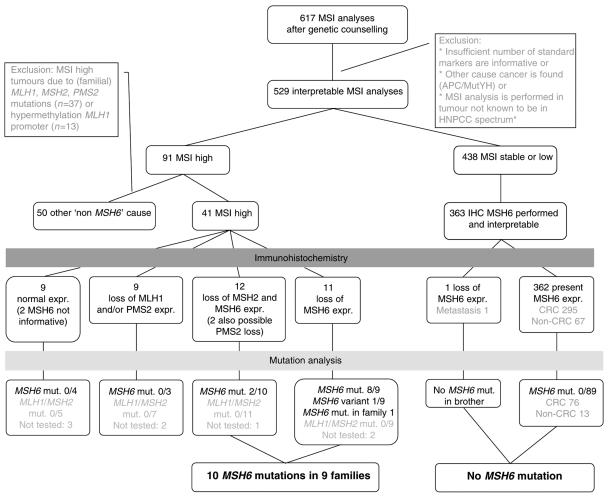
Flowchart MSI analyses. *HNPCC spectrum: CRC, endometrial cancer, sebaceous carcinoma, urothelial cell carcinoma, and brain tumour. *MSH6* mut: found pathogenic *MSH6* mutations *vs* number of patients in which *MSH6* was analysed; *MLH1/MSH2* mut: found pathogenic *MLH1/MSH2* mutations *vs* number of patients in which *MLH1* and *MSH2* were analysed; not tested: number of patients in which no mutation analyses were performed.

The study is based on 617 tumours of patients or their family members suspected of HNPCC that visited our clinical genetics department in which MSI and subsequent analyses were performed between 1997 until 2006 (Figure 1). In the families analysed in our study MSI analysis is performed in the tumour of the youngest relative available. All findings in this group that were available at 1-1-2006 are included in this study. In 529 tumours of patients a reliable distinction between MSI-high and MSI-stable/low could be made using the standard set of markers (Boland *et al*, 1998). IHC of MMR proteins became available and was applied for from 1999, in some cases retrospectively. IHC of the MSH6 protein was performed in all tumours regardless of MSI results. IHC of all MMR proteins was performed in case of an MSI- high or MSI-low tumour or when other tissue than CRC was tested, such as endometrial cancer, gastric cancer, sebaceous carcinoma, urothelial cell carcinoma, and brain tumours (Rodriguez-Bigas *et al*, 1997). We focused on two separate cohorts of patients; patients with and without MSI in their tumour DNA. The pedigrees made as a part of the genetic counselling procedure were studied for fulfilment of Amsterdam II criteria and Bethesda guidelines (Rodriguez-Bigas *et al*, 1997; Umar *et al*, 2004).

The study was performed according to the rules of the Medical Ethics Committee of the Radboud University Nijmegen Medical Centre.

### Molecular analysis

For MSI analysis normal and tumour tissues were extracted from formalin- fixed and paraffin-embedded tissues. The Bethesda microsatellite panel D2S123, D5S346, D17S250, BAT25, and BAT26 (Boland *et al*, 1998) was used essentially according to methods described previously (Hoogerbrugge *et al*, 2003). A tumour was considered MSI-high when instability was found in ⩾2 out of five markers (*n*=91) and MSI-stable or low in case of instability in ⩽1 out of five markers (*n*=438). In 178 samples the mononucleotide marker BAT40 was included in the standard marker set. IHC of the MMR proteins was performed with the monoclonal antibodies against MSH6 (Transduction lab code: G70220), MLH1 (Pharmingen code: 51–1327gr), PMS2 (Pharmingen code: 556415), and MSH2 (Oncogene code: NA26). Germline *MSH6* mutation analysis of the coding regions and splice sites of the *MSH6* gene was performed with a combination of sequence analysis (exon 1, splice acceptor site of exon 10), one-dimensional denaturing gradient gel electrophoresis (exons 2 up to and including 10) essentially as described by Wu *et al* (1999) and multiplex ligation-dependent probe amplification (MRC Holland) for the detection of exon deletions and duplications (exon 1 to 10). Only changes located within 10 nucleotides of the coding region that have not been described as polymorphisms before, are reported.

### Patients with an MSI-high tumour

*MSH6* germline mutation analysis was performed in a group of 19 patients with MSI-high HNPCC-associated tumours and loss of MSH6 expression in which *MLH1* and *MSH2* mutations were excluded. Nine of these tumours showed loss of MSH6 expression in the presence of MSH2 expression and 10 showed loss of both MSH2 and MSH6 expression, of which two were difficult to interpret and possibly also showed loss of PMS2 expression. Microsatellite instability patterns of HNPCC-associated tumours of 12 *MLH1*, 22 *MSH2*, and 10 *MSH6* mutation carriers were studied to compare the instability patterns of tumours of patients with germline mutations in *MSH6* to those with germline mutations in *MLH1* and *MSH2*.

### Patients with a non-MSI-high tumour

Three hundred and sixty-three non-MSI-high HNPCC-associated tumours (295 CRC) were analysed out of 335 families. Patients most suspected of HNPCC were selected by fulfilment of at least one of the following criteria; (1) age at diagnosis below 50 years, (2) first degree relative with an HNPCC-related tumour, or (3) second CRC. Of the patients that fulfilled one or more of these criteria a subgroup of 89 patients, 76 of whom had CRC, and one first degree relative, were analysed for *MSH6* germline mutations.

### Statistical analysis

Categorical variables were compared with the use of the Fisher's exact test using SPSS, version 12.0. A *P*-value of 0.05 is considered as threshold for statistical significance.

## RESULTS

### MSH6 mutation analysis in patients with an MSI-high tumour

In a group of 19 patients with both an MSI-high HNPCC-associated tumour and loss of MSH6 expression, but no detectable defect in *MLH1* or *MSH2*, 10 pathogenic mutations in *MSH6* were found in nine families (Table 1). Besides the nine different *MSH6* germline mutations found in patients with an MSI-high tumour, two pathogenic mutations in *MSH6* were found in patients in whom MSI analysis could not be performed. The mean age at diagnosis of the 11 index patients from the families with a pathogenic *MSH6* mutation was 44 years (range 36–57). The MSI analyses in nine of these index patients with an *MSH6* mutation was performed on four endometrial, four colorectal, and one urothelial cell cancer. All *MSH6* mutation carriers fulfil one or more Bethesda guidelines and in 64% of the families the Amsterdam II criteria are fulfilled. In the *MSH6* families endometrial cancers occur as frequently as CRCs.

Of the remaining nine tumours with loss of MSH6 expression, eight tumours also showed loss of MSH2 expression of which two were difficult to interpret and possibly showed loss of PMS2 expression as well, suggesting the presence of an as yet undetected *MSH2* (or *PMS2*) germline mutation. One tumour, a CRC developed at age 53, exclusively showed loss of the MSH6 protein. In this female patient an *MSH6* variant c.2117T>C (p.Phe706Ser) was found of which the pathogenicity is uncertain. She also carries a pathogenic mutation in *BRCA2* (c.3269del (p.Met1080fs)). The patients’ mother carries the same *MSH6* variant but not the *BRCA2* mutation. She was diagnosed with endometrial cancer at age 62. Microsatellite instability analysis and IHC on her tumour were inconclusive.

Stability in one or more of the dinucleotide markers occurred significantly more often in colorectal tumours of *MSH6* than of *MLH1* and *MSH2* mutation carriers (Table 2). Stability of mononucleotide markers is uncommon in tumours of *MSH6* as well as *MLH1* and *MSH2* mutation carriers.

### MSH6 mutation analysis in patients with a non-MSI-high tumour

Immunohistochemical staining showed MSH6 expression in all 295 non-MSI-high CRCs and in 67 out of 68 other non-MSI-high HNPCC-related tumours (Table 3).

A subgroup of patients with the highest suspicion of HNPCC, was tested for the presence of *MSH6* germline mutations. In none of the 76 patients with CRC, or in the 13 patients with other HNPCC-related tumours a pathogenic germline mutation in *MSH6* was detected. One non-MSI-high tumour of metastatic tumour tissue (most probably derived from a CRC) of a deceased patient showed loss of MSH6 expression, in presence of MLH1 and MSH2 expression. Because mutation analysis could not be performed in the deceased patient, mutation analysis in her brother was performed. No mutation in *MSH6* was detected (Table 4).

Silent variants c.3852G>A, c.2154C>T, c.1068T>C, and c.3246G>T were found. None of these are predicted to affect splicing and thus do not seem to have functional consequences. The missense variant c.3101G>C (p.Arg1034Pro) that was found in a female patient with CRC at age 43 might be pathogenic. As the carcinoma was not available the MSI and IHC analyses were performed in an adenoma, which might have decreased the sensitivity of the analyses. Segregation analysis in the family showed that her brother who had a glioma, and the mother who had two sisters with anamnestic endometrial cancer did not carry the *MSH6* variant, making the pathogenecity of this variant less likely.

## DISCUSSION

In this study, not one pathogenic germline *MSH6* mutation was detected in HNPCC suspected patients with a non-MSI-high CRC or HNPCC-related tumour.

Previous studies suggested that the sensitivity of MSI analysis to predict an *MSH6* mutation is low and that MSI should not be used as a selection criterion for *MSH6* mutation analysis (Wu *et al*, 1999), finding microsatellite stable or low patterns in 17% up to 50% (Berends *et al*, 2002; Hendriks *et al*, 2004; Plaschke *et al*, 2004; Niessen *et al*, 2006; Pinto *et al*, 2006) of HNPCC-associated tumours of *MSH6* mutation carriers. However careful consideration of previous studies is required as part of the conclusions are based on *MSH6* missense mutations of unknown pathogenecity or testing a sporadic tumour within an HNPCC family (a phenocopy) as suggested by positive immunostaining of MSH6 in the tumour. These have an unfavourable effect on the sensitivity of MSI analysis. In addition MSI analysis on endometrial cancer, the most frequent tumour in female *MSH6* mutation carriers might decrease its sensitivity, as it is known that the instability in these tumours is generally less pronounced (Wijnen *et al*, 1999; Hendriks *et al*, 2004).

*MSH6* mutations result in a weaker mutator phenotype (Kolodner *et al*, 1999), which may be explained by the major function of *MSH6* to correct base–base mismatches and single nucleotide deletion loops but not larger deletion loops (Parc *et al*, 2000). Like in previous studies (Kolodner *et al*, 1999; Verma *et al*, 1999; Parc *et al*, 2000) our study shows that mononucleotide markers but not dinucleotide markers are sensitive to show instability in tumours of *MSH6* mutation carriers. The sensitivity of MSI analysis therefore depends on the microsatellite markers used. Enlarging the standard (Bethesda) marker set (Boland *et al*, 1998) with a mononucleotide marker (like BAT40) will increase the sensitivity of MSI analysis by minimising the chance of missing tumours with MSH6 inactivation. As data on MSI analysis of other non-colorectal HNPCC-related tumours with defective MMR are insufficient, we recommend additional IHC of MLH1, PMS2, MSH2, and MSH6 proteins when MSI analysis is performed on non-colorectal HNPCC-related cancers. Immunohistochemical staining of MMR proteins will also improve the interpretation of MSI patterns when a low percentage of tumour cells or an adenoma is tested or when only one mononucleotide marker shows instability (MSI low). When a patient is excluded from further HNPCC analysis based on a non-MSI-high pattern in tumour DNA, a second MSI analysis in the family should always be considered to avoid missing a germline mutation because of an initial test in a phenocopy.

From previous studies we know, that in *MSH6* mutation carriers CRC occurs at older age than in *MLH1* and *MSH2* mutation carriers (Hendriks *et al*, 2004). In our study, the patients with MSI-stable/low tumours that were analysed for *MSH6* mutations were mainly diagnosed before the age of 50. This selection is not expected to have a large influence, because MSI analysis in the families in our study is performed in the tumour of the youngest relative available. The mean age of diagnosis in *MSH6* mutation carriers is above 50, but the occurrence of one relative below 50 is expected to be present in most of the *MSH6* families. The pedigrees of the diagnosed *MSH6* families in our study all contained an affected relative diagnosed below 50 years of age.

The prevalence of *MSH6* mutation carriers in HNPCC suspected CRC patients is low, as is demonstrated by the fact that we detected an *MSH6* mutation in only about 1% of these patients. All these mutations were found in patients with an MSI-high tumour. Data from previous studies (Berends *et al*, 2002; Hendriks *et al*, 2004; Plaschke *et al*, 2004; Barnetson *et al*, 2006; Niessen *et al*, 2006) show that approximately 15% of colorectal tumours of *MSH6* mutation carriers do not have an MSI-high pattern, whereas they do show loss of MSH6 expression and thus might be the result of the *MSH6* germline mutation. On the other hand, approximately 5% of colorectal tumours of *MSH6* mutation carriers do show neither an MSI-high pattern nor loss of MSH6 expression and thus might have arisen independent from the genetic background of the carrier. Based on our finding of the low incidence of *MSH6* mutations in HNPCC-suspected CRC patients and the percentage of non-MSI-high tumours in MSH6 mutation carriers from the literature, the probability of missing a mutation by not performing mutation analyses in patients with non-MSI-high CRCs is expected to be extremely low. This is confirmed by the fact that we did not find any non-MSI-high CRC with loss of MSH6 expression, nor a germline *MSH6* mutation in any of the patients with a non-MSI-high tumour. Our findings show that MSI analysis is highly suited to trace CRC of carriers of *MSH6* germline mutations.

## Figures and Tables

**Table 1:** Characteristics of patients with a germline mutation in *MSH6*

		**MSI high**								
**Tested cancer and age at diagnosis**	**Pathogenic mutation *MSH6***	**Instable mono-nucleotides**	**Instable di-nucleotides**	**IHC *MSH6***	**IHC *MSH2***	**Amsterdam criteria II**	**Bethesda A**	**Bethesda B**	**EN in family**	**CRC in family**
CO 42	c.265−?_457+?dup	2/3	2/3	Neg	Pos	+	−	+	+	+
EN 57^a^	c.814G>T (p.Glu272X)	3/3	0/3	Neg	Pos	+	−	+	+	+
CO 52^b^	c.651dup (p.Lys218X)	2/2	3/3	Neg	Pos	+	+ EN 37	+	+	+
*CO 58* b	*c.651dup (p.Lys218X)*	*3/3*	*2/3*	*Neg*	*Neg*	+	+ *CO 60*	+	+	+
EN 36	c.3838C>T (p.Gln1280X)	2/2	1/3	Neg	Pos	+	−	+	+	+
CO 50	c.3273dup (p.Lys1092X))	2/2	2/3	Neg	Pos	+	+CO 46/CO 50	+	+	+
EN 43	c.3261dup (p.Phe1088fs)	2/2	3/3	Neg	Neg	+	−	+	+	−
CO 39	c.3261del (p.Phe1088fs)	2/2	2/3	Neg	Pos	+	−	+	−	+
EN 38	c.1135_1139del (p.Arg379X)	2/2	1/3	Neg	Pos	−	+O 38	−	+	−
UR 56	c.1−?_475+? del	3/3	0/3	Neg	Pos	−	+UR 57SEB 59	+	−	+
EN 38	c.3678_3706dup (p.Ala1236fs)	nt		nt	nt	−	−	+	+	−
CO 47	c.2815C>T (p.Gln939X)	nt^c^		nt	nt	−	−	+	−	+
Total						7/11	4/11	10/11	8/11	8/11
						(64%)	(36%)	(91%)	(73%)	(73%)

*Bethesda A*: Proband with two HNPCC-related cancers, *Bethesda B*: Proband and first degree relative with HNPCC-related cancer, one diagnosed <50 y.

EN=endometrial cancer, CO=colorectal cancer, UR=urothelial cell carcinoma, SEB=sebaceous adenoma, O=ovarian cancer, Neg=negative, Pos=positive, nt=not tested; IHC=immunohistochemistry; MSI=microsatellite instability.

a This patient also has an UV c.65G>C (p.Gly22Ala) in *MLH1*

b Patients from same family.

c Tumour of patients father showed MSI and no *MSH6* expression.

**Table 2:** Results of the MSI analysis in *MSH6*, *MLH1* and *MSH2* mutation carriers

**MSI pattern**	***MSH6*** **mutation carriers**	***MLH1* and *MSH2* mutation carriers**	***P*-value Fisher exact**
*One or more of three dinucleotides*^a^ *stable*			
CRC	4/5 (80%)	4/22 (18%)	**0.017**
Non CRC	4/5 (80%)	1/6 (17%)	NS
			
*One or more mononucleotides*^b^ *stable*			
CRC only	1/5 (20%)	2/26 (8%)	NS
Non CRC	0/5 (0%)	0/6 (0%)	NS

NS=not significant; CRC=colorectal cancer; MSI=microsatellite instability.

a D2S123, D5S346, and D17S250.

b BAT25 and BAT26.

**Table 3:** Overview of microsatellite stable/low tumours

	**Patient with non-MSI-high tumour and loss of MSH6 expression**	**Patients with non-MSI-high tumours and positive MSH6 expression**	**Selected group of patients with non-MSI-high tumours and positive MSH6 expression without a pathogenic mutation in *MSH6***
***Colorectal ca***		295	76
Age <50 yr		171 (58%)	62 (82%)
***Other HNPCC-related neoplasia***	1	67	13
Endometrial ca		15	3
Gastric ca		3	
Sebaceous ca		4	
Urothelial cell ca		1	
Brain tumour		1	
Metastatic tissue	1^a^	7	
Small bowel		1	
Adenoma			
Colon		34	10
Duodenum		1	
Age <50 yr	0	34 (51%)	9 (69%)

MSI=microsatellite instability; HNPCC=hereditary non-polyposis colorectal cancer; ca=cancer.

a Mutation analysis in the patients’ brother showed no *MSH6* mutation.

**Table 4:** MSI-test result and IHC protein expression pattern of tumours from patients tested for the presence of a *MSH6* germline mutation

**MSI**		**MSI high**		**MSI stable/low**	
IHC	MSH6−	MSH6−	MSH6−a	MSH6−	MSH6+
	MSH2+	MSH2-	MSH2−a		
	MLH1+	MLH1+	MLH1+^a^		
	PMS2+	PMS2+	PMS2−a		
No pathogenic mutation in MSH6	1^b^	6	2	1^c^	89
Pathogenic mutation in MSH6	8	2			

IHC=immunohistochemistry; MSI=microsatellite instability.

a IHC difficult to interpret.

b With MSH6 variant c.2117T>C (p.Phe706Ser).

c Mutation analysis was performed in the patients’ brother.
